# Enteric Neuronal Damage, Intramuscular Denervation and Smooth Muscle Phenotype Changes as Mechanisms of Chagasic Megacolon: Evidence from a Long-Term Murine Model of *Tripanosoma cruzi* Infection

**DOI:** 10.1371/journal.pone.0153038

**Published:** 2016-04-05

**Authors:** Camila França Campos, Silvia Dantas Cangussú, Ana Luiza Cassin Duz, Christiane Teixeira Cartelle, Maria de Lourdes Noviello, Vanja Maria Veloso, Maria Terezinha Bahia, Camila Megale Almeida-Leite, Rosa Maria Esteves Arantes

**Affiliations:** 1 Departamento de Patologia Geral Instituto de Ciências Biológicas, Universidade Federal de Minas Gerais, Belo Horizonte, Minas Gerais, Brazil; 2 Departamento de Ciências Biológicas/LAFEX, Universidade Federal de Ouro Preto, Ouro Preto, Minas Gerais, Brazil; 3 Departamento de Ciências Biológicas/NUPEB, Universidade Federal de Ouro Preto, Ouro Preto, Minas Gerais, Brazil; 4 Departamento de Morfologia, Instituto de Ciências Biológicas, Universidade Federal de Minas Gerais, Belo Horizonte, Minas Gerais, Brazil; Albert Einstein College of Medicine, UNITED STATES

## Abstract

We developed a novel murine model of long-term infection with *Trypanosoma cruzi* with the aim to elucidate the pathogenesis of megacolon and the associated adaptive and neuromuscular intestinal disorders. Our intent was to produce a chronic stage of the disease since the early treatment should avoid 100% mortality of untreated animals at acute phase. Treatment allowed animals to be kept infected and alive in order to develop the chronic phase of infection with low parasitism as in human disease. A group of Swiss mice was infected with the Y strain of *T*. *cruzi*. At the 11^th^ day after infection, a sub-group was euthanized (acute-phase group) and another sub-group was treated with benznidazole and euthanized 15 months after infection (chronic-phase group). Whole colon samples were harvested and used for studying the histopathology of the intestinal smooth muscle and the plasticity of the enteric nerves. In the acute phase, all animals presented inflammatory lesions associated with intense and diffuse parasitism of the muscular and submucosa layers, which were enlarged when compared with the controls. The occurrence of intense degenerative inflammatory changes and increased reticular fibers suggests inflammatory-induced necrosis of muscle cells. In the chronic phase, parasitism was insignificant; however, the architecture of Aüerbach plexuses was focally affected in the inflamed areas, and a significant decrease in the number of neurons and in the density of intramuscular nerve bundles was detected. Other changes observed included increased thickness of the colon wall, diffuse muscle cell hypertrophy, and increased collagen deposition, indicating early fibrosis in the damaged areas. Mast cell count significantly increased in the muscular layers. We propose a model for studying the long-term (15 months) pathogenesis of Chagasic megacolon in mice that mimics the human disease, which persists for several years and has not been fully elucidated. We hypothesize that the long-term inflammatory process mediates neuronal damage and intramuscular and intramural denervation, leading to phenotypic changes in smooth muscle cells associated with fibrosis. These long-term structural changes may represent the basic mechanism for the formation of the Chagasic megacolon.

## Introduction

Chagas’ disease (CD) is caused by the flagellate protozoan *Trypanosoma cruzi* and is transmitted by hematophagous insects of the subfamily Triatominae [1]. It is a clinical and polymorphic entity and a major cause of morbidity and mortality. The first cases of CD were reported 9000 years ago [2]. Earlier estimates indicate that approximately 10 million people are infected worldwide [3], and after the implementation of vector control, disease prevalence decreased in many endemic areas [4,5]. However, long-term epidemiological surveillance was not maintained, and limited surveillance may have allowed the reinfestation by triatomine insects [6,7]. At present, approximately 25 million individuals are exposed to infection and the disease has an annual incidence of 200,000 new cases [3,8].

Human trypanosomiasis is characterized by two clinically distinct stages. The acute phase has a short duration of approximately two months and can be asymptomatic or symptomatic [9]. After the acute phase, the patient progresses to the chronic phase, with different clinical manifestations, from the lack of symptoms to severe cardiac or gastrointestinal (GI) disorders. Approximately 70% of the infected individuals remain asymptomatic [10] in the indeterminate phase of the disease, which can last two or more decades [11]. The chronic digestive form, which is the focus of our study, can involve the presence of megaesophagus and megacolon in severe human cases [9,12,13]. These conditions are characterized by significant morphological changes, including dilatation and hypertrophy of the intestinal wall, and physiological disorders that affect predominantly the sigmoid region, with loss of the organ’s motor coordination [14]. However, the pathogenic mechanisms of megacolon have not been fully elucidated.

The GI tract is controlled by the enteric nervous system (ENS), including parasympathetic neurons and glia [15], and by extrinsic sympathetic and parasympathetic fibers, which control the smooth muscles via myenteric plexus to contract or relax the sphincter and either inhibit (via sympathetic regulation) or stimulate (via parasympathetic regulation) motility and secretion [16,17]. It has been postulated that the relationship between neuronal loss in the myenteric plexus during the acute phase [10,12,18] and changes in contractility and tonus of the muscular layer [10,12] could determine symptoms, although no pathogenetic explanations are widely accepted. We believe that the denervation of the myenteric plexus [10,12] and the formation of areas of fibrosis [19] can modify the architecture of the smooth muscles and alter the intermuscular nerve fibers, and these processes are responsible for classically described symptoms, including dysphagia, odynophagia, retrosternal pain, and progressive constipation [3]. Any further disturbance in the peristaltic movement will result in stagnation and retention of GI tract contents, with consequent distension of the muscle fibers, leading to hypertrophy. These changes will lead to mega formations, which are outcomes of the intrinsic innervation disorder [12] found in chronic digestive CD. Patients with megacolon have rates of denervation in the myenteric plexus exceeding 55%, in addition to hypertrophy (63% increase in diameter) of the remaining neurons [18].

As far as we know, no studies have addressed the changes in the intramuscular innervation in human CD. In fact, human CD is very complex and no valid chronic experimental model has been established to date. It is very difficult to study the digestive form of human CD because of the limited knowledge of the pathology in the acute and chronic phases owing to the lack of samples from humans. Consequently, much of the knowledge has been obtained from experimental models that do not offer a definitive pathogenetic explanation for the phenomena [20,21,22] primarily because the duration of the chronic phase in those models is too short and mimics the human acute phase only. Indeed, murine models have been the target of several studies owing to the facility of obtaining, maintaining, and handling the experimental animals. Several parameters have been analyzed, including the parasite-host relationship, behavior of *T*. *cruzi* strains, drug effectiveness, and the immune response and histopathology of the host [23–25]. However, these studies are restricted to the evaluation of changes in the acute phase owing to the high mortality of mice after the peak of parasitemia. Other studies have investigated aspects of motility disorders in T. cruzi infected mice. Dilatation of the intestines [26] and decrease in intestinal motility was observed in mice infected by different groups of T. cruzi [27,28]. Moreover, selenium-enriched chow was able to prevent decrease in intestinal motility in infected mice [29].

According to Arantes *et al*. [30], in C57BL/6 mice, the denervation of the myenteric plexus during the acute phase appears to be one of the most relevant histological changes observed in the mouse colon. In addition to the parasitism and intense inflammatory infiltrate in the intestinal wall, changes are observed in the enteric glia and extrinsic innervation. All these changes and their outcomes in the chronic phase have not been systematically studied in humans and only a few studies have evaluated these aspects in murine models [22,24]. We hypothesized that long term chronically infected animals would present the structural modification of the colon wall being useful as a model to understand the pathogenic mechanisms of chagasic megacolon. Therefore, the aim of this study was to develop a murine model that reproduces, for the first time, the long-term and dynamic morphofunctional changes of the chronic phase of human CD in the GI tract.

This model allows the evaluation of histopathological changes in the intestinal wall and in the intramural ENS in both acute and chronic phases. In addition, we showed for the first time that denervation includes not only the inflammatory-induced loss of ganglion cell bodies but also important changes in the innervation pattern of the muscular layers, as already suggested in experimental colitis [31]. We believe that this animal model of chronic CD may be a useful tool to study the mechanism of mega formation in this neglected disease. In addition, it can be used to explore potential therapeutic targets to control Chagas’ disease manifestations and to study other intestinal inflammatory neuromuscular disorders.

## Materials and Methods

### Ethics statement

Animal experiments were performed in strict accordance with the Regulations for the Administration of Affairs Concerning Experimental Animals [Law number 11.794 (October 8^th^, 2008) and resolution number 15/2007 (September 6^th^, 2007)]. The protocol was approved by the Institutional Animal Care and Use Committee (Comitê de Ética em Experimentação Animal- CETEA) of the Universidade Federal de Minas Gerais (UFMG) for the use of laboratory animals (Permit numbers 222/2007 and 377/2012). The ethics committee of UFMG specifically reviewed and approved the mortality aspects of this study protocol. Euthanasia was performed under high doses of pentobarbital and all efforts were made to minimize suffering.

### Animals

Fifty-six 4-week-old female Swiss mice were supplied by the Animal Facility of UFMG and maintained in a temperature- and light-controlled room with access to food and water *ad libitum*. Minimum possible suffering was guaranteed by daily monitoring of animals until 11^th^ day. After that the treated animals and non-infected controls were monitored each two days until 20^th^ day. By the rest of the experimental period, the animals were monitored weekly. They were weighted before infection and at the day of euthanasia.

### Infection and induction of Chagasic megacolon

Ten mice were used as non-infected controls, of which five animals composed the acute-phase control group and five were used as the chronic-phase control group. The other 46 animals were inoculated intraperitoneally (i.p.) with 50.000 blood trypomastigote forms of the Y strain of *T*. *cruzi*. For preparation of the *inoculum*, parasites were counted according to the technique described by Brener [32] and provided by Dra. Maria Terezinha Bahia, from Laboratório de Doença de Chagas do Instituto de Ciências Exatas e Biológicas, Universidade Federal de Ouro Preto (UFOP). After confirming the infection of all inoculated animals with a fresh blood test, the animals were divided into two groups: (1) acute-phase infected group: 10 animals were destined for euthanasia on the 11^th^ day after infection (d.a.i.), and (2) chronic-phase infected group: on the 11^th^ d.a.i., 30 animals were treated with a single oral dose of benznidazole (Rochagan® Roche, Rio de Janeiro, Brazil) at 500 mg/kg of body weight and were reared until 15 months after infection (m.a.i.). During this period 80% of infected and treated animals died spontaneously. It was not possible to use humane endpoints because these animals did not showed signs of sickness, pain, distress, suffering or moribund conditions. The surviving animals were euthanized at the end of the experiment. Another group of 6 animals was maintained to determine the rate of parasitemia until the 15^th^ day when were euthanized. The described protocol was entirely repeated twice.

### Evaluation of anti-*T*. cruzi IgG levels

Blood samples from treated and infected mice were collected from the orbital venous sinus (500 μL) at 15 months after infection. *T*. *cruzi*-specific antibodies were detected using the technique described by Voller [33]. Enzyme-linked immunosorbent assay (ELISA) plates were coated with *T*. *cruzi* antigen prepared from the alkaline extraction of the Y strain at exponential growth in LIT medium. Anti-mouse IgG peroxidase-conjugated antibody (Sigma Chemical Co.) was used. The mean absorbance for 10 negative control samples plus two standard deviations was used as the cut-off to discriminate positive and negative results.

### Histopathology analysis

As described by Arantes and Nogueira (1997) [34], the whole intestine was separated from the mesentery, washed in PBS (phosphate buffer saline at 0.01 M, pH 7.3), and extended to serosa in contact with the filter paper. The antimesenteric border was opened and all its contents were removed without damaging the mucosa. Subsequently, the length of each colon fragment was measured (cm). These fragments were immersed in Bouin solution with 2% glacial acetic acid during 10 minutes. The pre-fixed colon was rolled into a spiral with the mucosa facing inward to form rollers from the distal portion (anus) to the proximal portion (cecum), with adaptations of the technique described by Calvert et al. (1989) [35].

The colon Swiss rolls were fixed in 10% buffered formalin solution, dehydrated, cleared, and embedded in paraffin. The paraffin blocks were cut into 4-μm sections and stained with hematoxylin-eosin (H&E) for assessment of inflammation, Gomori’s trichrome stain for the quantitative evaluation of fibrosis, and with silver Gomori stain for visualization of the reticular fibers.

### Immunostaining

Tissue sections were stained by immunohistochemistry with the following antibodies: anti-*T*. *cruzi* to detect tissue parasitism [36], anti-iNOS to detect the presence of inducible NO synthase, anti-PGP 9.5 to evaluate the morphology of the nervous tissue, and anti-GAP 43 to evaluate the density of reinnervation. Sections from the colonic segments were subjected to blockade of endogenous peroxidase in 100 ml PBS at 10% and 3.5 ml of hydrogen peroxide at 30% for 30 minutes. Subsequently, these sections were incubated with anti-*T*. *cruzi* antibody (1:5000, obtained from rabbits inoculated with blood trypomastigote forms of the Berenice-78 strain of *T*. *cruzi* provided by Dr. Maria Terezinha Bahia), anti-iNOS antibody (1:500; polyclonal rabbit anti-nitric oxide synthase II, AB5382, Millipore, USA), anti-PGP 9.5 antibody (1:500; polyclonal rabbit anti-protein gene product 9.5, RA95101, UltraClone Ltd, England), or GAP 43 (1:500; polyclonal rabbit anti-growth-associated protein-43, AB5220, Chemicon, United States) diluted in PBS/1% bovine serum albumin (1870, Inlab, Diadema, Brazil) at 4°C for 15 hours. Subsequently, the tissue sections were incubated with the secondary anti-rabbit antibody and biotinylated anti-mouse diluted pre-Kit (Dako, LSAB, K0675) in a moist chamber for 30 minutes at room temperature. After bathing in PBS, the sections were incubated in streptavidin-peroxide diluted pre-Kit (Dako, LSAB, K0675) in a moist chamber for 30 minutes at room temperature. Peroxidase activity was detected after incubating the sections in 50 mg DAB solution (3,3'-diaminobenzidine tetrahydrochloride) (Sigma, St. Louis, MO) for 5 minutes.

### Morphometry analysis

The optical microscope images were captured with a resolution of 1392 × 1040 pixels and transferred from a video color camera (Cool/Snap Proof Color; Media Cybernetics, Bethesda, MD, USA) to a video system attached to a computer using the program Image-Pro Express version 4.0 for Windows (Media Cybernetics, Bethesda, MD, USA). The images were analyzed using the KS300 program (Zeiss, Jena, Germany) in the Laboratory of Morphometry of the Department of General Pathology of the Institute of Biological Sciences.

Body mass and colon length were measured immediately after euthanasia. The parameters measured were thickness of the total colon wall (μm), thickness of the total muscular layer (μm), and area of the muscle cells (μm^2^) (calculated as the field area/number of smooth muscle cells).

The stained area per total area (μm^2^) was measured for anti-GAP43, Gomori’s trichrome, and silver Gomori staining. For the anti-iNOS antibody, only qualitative analysis was performed.

To evaluate the myenteric plexus (intermuscular innervation), the area of the total myenteric ganglia and the area positive for PGP 9.5 inside the ganglia were measured. The total number of cells in the myenteric plexus and PGP-stained neuronal cells were counted. The ratio between these variables was represented as *PGP 9*.*5–stained area/myenteric plexus* total area and *PGP 9*.*5-stained neuronal cell number/total number of ganglion cells*. The anti-PGP 9.5-stained area per total muscle area, excluding the myenteric plexus, was also measured to evaluate the density of intramuscular innervation. We analyzed an average of 13 fields/intestine in areas affected by intense and visible inflammation. The number of parasites immunostained with anti-*T*. *cruzi* serum was counted. We analyzed 10 fields per animal at a 40× magnification for each parameter (total area of 238.336 μm^2^).

### Data analysis

The results were expressed as the average and standard deviation (SD) or median of two independent experiments. The degree of significance was calculated using one-way ANOVA followed by Tukey’s post hoc test for normally distributed data or Kruskal-Wallis and Mann–Whitney (Wilcoxon) tests for non-parametric data. Probability values (p) of 0.05 or less were considered significant.

## Results

### Evaluation of infection and tissue parasitism

**Fig 1:**
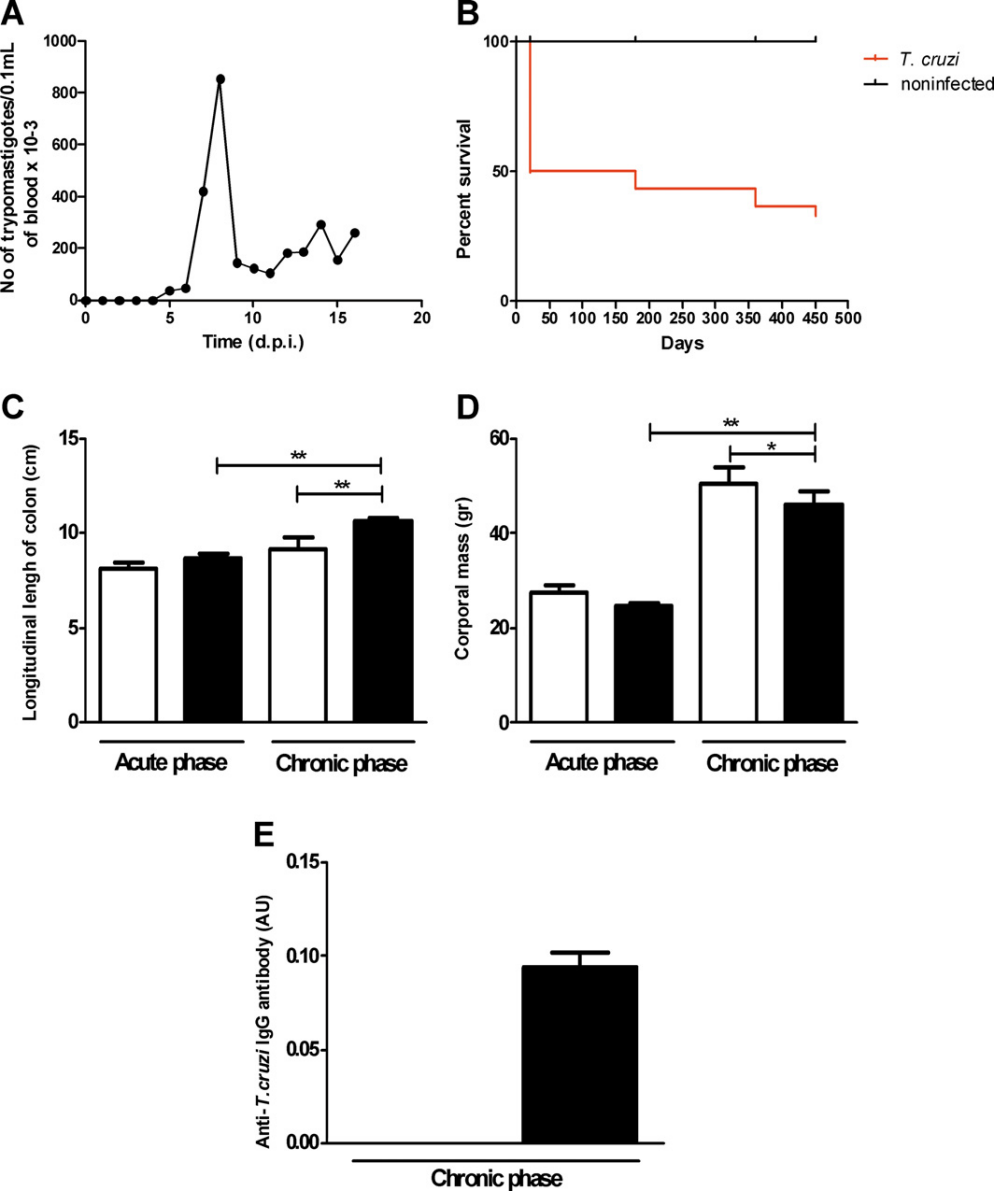
Infection parameters of Swiss mice infected with 50,000 trypomastigotes of the Y strain of *T*. *cruzi*. (A) The peak of parasitemia was at the 9^th^ d.a.i. (B) The mortality rate until the 20^th^ d.a.i. was significantly high, with death of approximately 50% of the animals treated with benznidazole. (C) The body mass of the chronic-phase infected group decreased compared with the control group but increased compared with the acute-phase infected group. (D) Colon length increased in the chronic-phase infected group compared with the control group of paired animals and with the acute-phase infected group. (E) ELISA of *anti-T*. *cruzi* total IgG was negative in the acute-phase infected group and positive in the chronic-phase infected group. Values represent the means ± SEM. *p = 0.023, **p = 0.001, N = 10 for each experimental and control group. The results represent two independent experiments. Black: infected groups; white: control groups.

The parasitemia curve showed a profile characteristic of infection with the Y strain with a peak at 9 d.a.i. (853 trypomastigotes per 0.1 mL of blood × 10^3^; Fig 1A). Mice infected with the Y strain showed 100% mortality during the acute phase between 14 and 18 d.a.i. (Fig 1B). On the 11^th^ d.a.i., 30 animals were treated with a single dose of benznidazole at 500 mg/kg of body weight and were reared until 15 m.a.i. (chronic-phase infected group). Until the 20^th^ d.a.i., the chronic-phase infected animals showed a significantly high mortality rate, with spontaneous death of approximately 50% of the animals during this period. By the 15^th^ m.a.i., 20% (6 animals) of the animals remained alive (Fig 1B), were euthanized and were used in the analysis.

The longitudinal length of the colon was longer in the chronic-phase infected group (10.6 cm) compared with the control group of paired animals (8.5 cm) and with the acute-phase infected group (8.6 cm), indicating dolichocolon (Fig 1D).

### Histopathological characterization of the inflammatory infiltration in the colon

#### Acute phase

**Fig 2:**
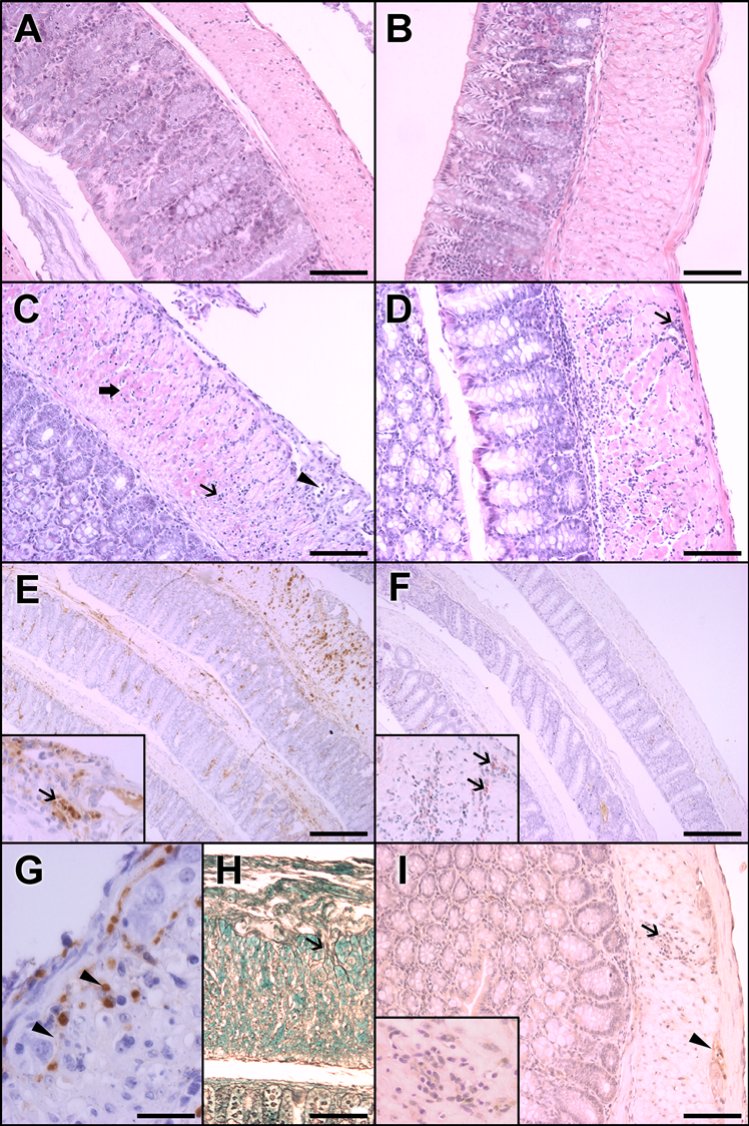
Histopathological features and evidence of parasitism in the colons of Swiss mice infected with 50,000 trypomastigotes of the Y strain of *T*. *cruzi*. A, C, E, G, and H represent acute-phase aspects (11 d.a.i.). B, D, F, and I represent chronic-phase aspects (15 m.a.i.). (A) The colons of the control group of paired animals in the acute phase present normal cellularity and thickness. (B) In the chronic phase, no alterations were observed in the control group. (C) In acute-phase infected animals, a mononuclear inflammatory infiltrate was observed in the submucosal and muscular layers (arrow). In the myenteric plexus (arrowhead) and in the inner muscular layer, there is evidence of muscle fiber necrosis (thick arrow). This transmural pattern is merged with non-inflamed areas (not shown). (D) In contrast, in the chronic-phase infected group, mononuclear infiltrates are focal and more intense in the outer muscular layer in the periganglionar and perivascular areas (arrow). (E) Intense parasitism (inset,arrow) is associated with inflammatory infiltrates in the acute phase. (F) In the chronic phase, parasites are scarce and not associated with inflammatory foci (inset). (G) The acute-phase infected group showed strong iNOS positivity associated with inflammatory and degenerative changes in the myenteric plexus (arrowheads) compared (I) with the weak staining in inflammatory cells (arrow, and inset) in the chronic-phase infected group. A possible glial cell of ganglia (arrowhead) is also stained. (H) In addition, a greater amount of reticular fibers with thickened areas around the ganglia (arrow) were present in the acute phase. The results represent two independent experiments. A, B, C, D, I, H Magnification at 20x. Scale bar corresponds to 20 μm. E, F Magnification at 4x. Scale Bar corresponds to 100 μm. G Magnification at 40x. Scale bar corresponds to 10 μm. HE staining in A, B, C and D. Immunohistochemistry with anti-*T*. *cruzi* antibody in E, and F, and insets of E and F. Immunohistochemistry with anti-iNOS antibody in G and I. Silver Gomori staining in H.

In the acute-phase infected group, the mucosal layer was discontinuous and characterized by desquamation of the epithelium due to necrosis or apoptosis and consequent decreased layer thickness. The submucosal layer was enlarged with edema, inflammatory cells (primarily mononuclear cells), vessels with endothelial swelling and congestion, and perivascular infiltrate (Fig 2C). A mild to intense parasitism with amastigote nests was observed throughout the extent of the muscular layers, particularly in regions near the vessels of the submucosal layer (Fig 2E).

It is of note that the presence of amastigote nests in and around the myenteric plexus is often associated with signs of necrosis or suggestive of neuronal degeneration in the cytoplasm (vacuolated) and nucleus (Fig 2E and inset).

The inflammatory infiltrate observed in all intestinal layers was predominantly mononuclear and more intense in muscular and submucosal layers. In the acute phase, infiltrates were found in the muscular layer throughout the extension of the colon, with the formation of bundles arranged between the muscle fibers, and these infiltrates were associated with extensive edema. Some isolated or small groups of fibers exhibited necrosis (Fig 2C).

In the acute phase, we detected an increase in the stained area indicating reticular fibers in the inner muscular layer compared with the control group due to enlargement of reticular fibers in tissue and around ganglia (Fig 2H).

#### Chronic phase

The mucous layer in the control animals that remained alive for the entire 15-month study period was slightly thinner than that in the control animals euthanized at the 11^th^ day.

The number of isolated amastigotes or nests significantly decreased in all sections of the colon in the chronic phase, as revealed by immunohistochemistry analyses using anti-*T*. *cruzi* antibody in animals in the acute (Fig 2E and inset) and chronic phases of infection (Fig 2F and inset). In cases in which parasitemia was absent in the chronic phase, we confirmed the absence of infection by the measurement of anti-*T*. *cruzi* IgG antibody using ELISA (Fig 1E).

Some chronic-phase animals presented foci of inflammatory infiltrates around some myenteric ganglia, and discrete parasitism in the submucosal and muscular layers (Fig 2F and inset, arrows,) whereas others did not have any nested or isolated amastigotes. No parasites were found in the myenteric plexus in the chronic-phase animals.

The inflammatory infiltrates in the submucosal layer ranged from mild to moderate, were mostly perivascular, and contained homogeneous mononuclear cells (Fig 2D). There was increased thickness of the vessel walls and hypertrophy of the endothelium, which suggests chronic vasculitis. In the muscular layer, discrete inflammatory infiltrates were observed across the inner muscular layer in most cases, between the inner and outer muscular layers.

### Increased thickness of the colon wall and fiber hypertrophy in the chronic-phase animals

**Fig 3:**
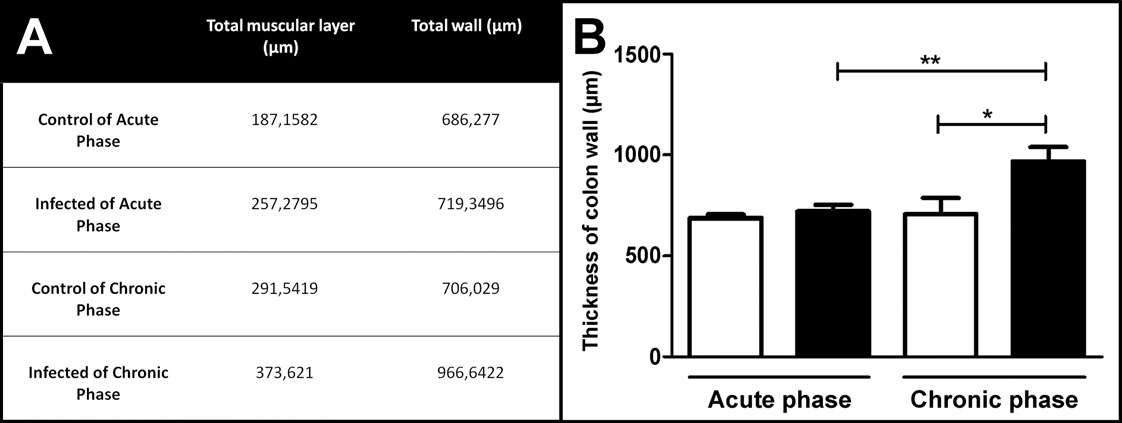
Morphometric evaluation of colon wall thickness in Swiss mice in the acute and chronic phases of infection with 50,000 trypomastigotes of the Y strain of *T*. *cruzi*. (A and B) The chronic-phase infected group presented increased thickness of the total colon wall compared with the acute-phase infected group and with the control group. Values represent means ± SEM. *p = 0.001, **p = 0.026. N = 10 for each experimental and control group. The results represent two independent experiments. Black: infected groups; white: control groups.

During the acute phase, no increase in the total colon wall thickness was observed (Fig 3A and 3B). However, the thickness of the total muscular layer increased in the acute-phase group compared with the non-infected paired control animals (Fig 3A).

The thickness of the total colon wall in the chronic-phase infected group increased (Fig 3A and 3B) compared with the control group and with the acute-phase infected group. Also, the thickness of the total muscular layer in the chronic-phase group increased compared with the control and acute-phase groups, indicating experimental Chagasic megacolon (Fig 3A).

**Fig 4:**
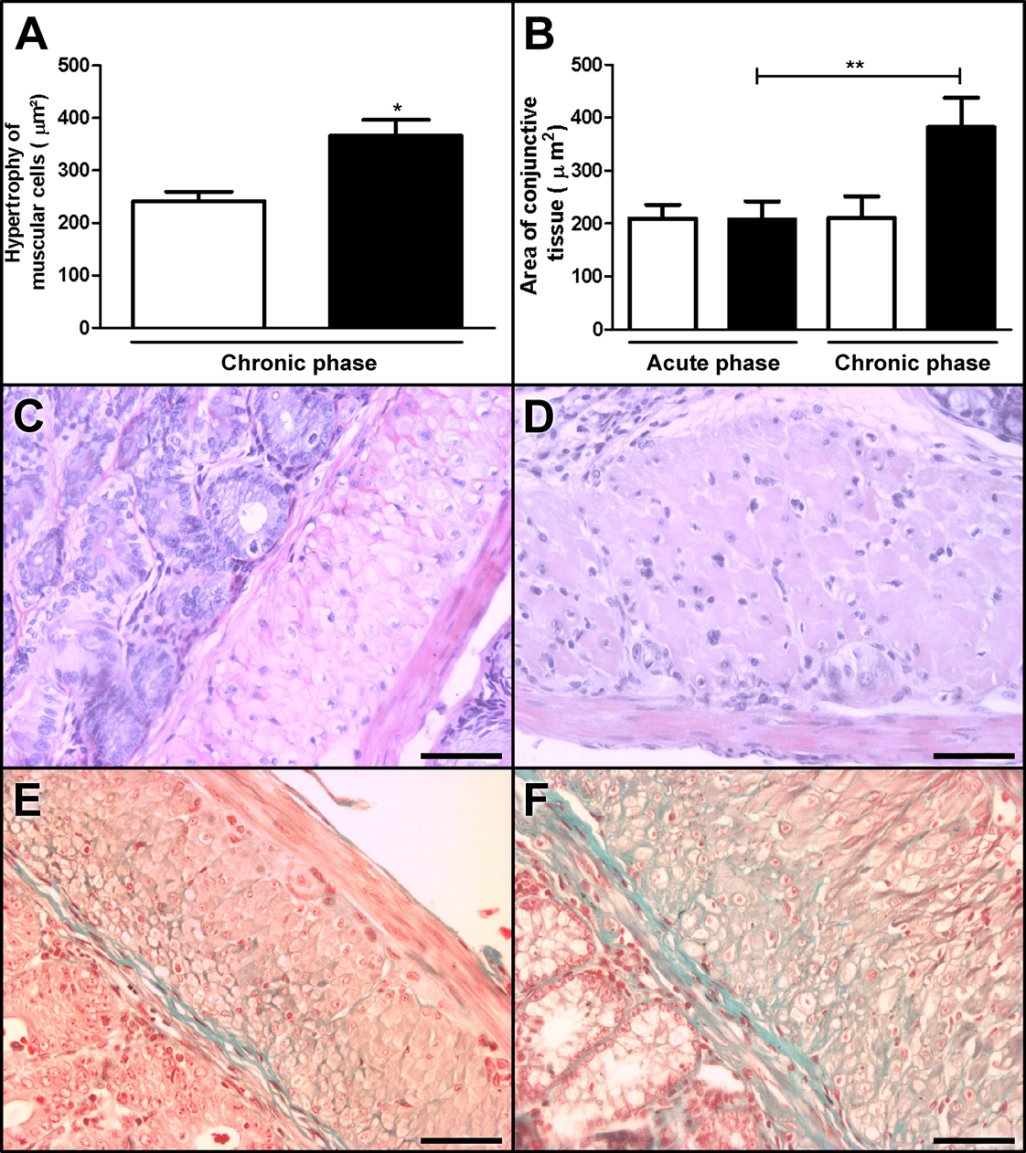
**Hypertrophy (A) and fibrosis (B) in the colons of chronic-phase Swiss mice infected with 50,000 trypomastigotes of the Y strain of *T*. *cruzi* Y strain**. (A) The volume of the muscle cells increased in the chronic-phase infected group (D) compared with the control group (C). (B) In addition, the amount of the collagen fibers located between the muscular fibers increased in the chronic-phase infected group (F) compared with the acute-phase infected group (E). Values represent means ± SEM. *p = 0.015, **p = 0.018. N = 10 for each experimental and control group. The results represent two independent experiments. Black: infected groups; white: control groups. C, D, E, and F Magnification at 20×. Scale bar corresponds to 20 μm. HE staining in C and D. Gomori’s trichrome staining in E and F.

There was no difference in the size of the muscle fibers of the inner and outer layers between the control and acute-phase animals. However, the volume of the fibers of the inner muscular layer was significantly larger in the chronic-phase group compared with the control animals, indicating hypertrophy of the muscle cells (Fig 4A, 4C and 4D).

### Increased fibrosis in the chronic-phase group

In the chronic-phase group, we observed a significant increase in the area of conjunctive tissue (382.480 μm^2^; Fig 4F) compared with the acute-phase group (207.760 μm^2^; Fig 4E) as revealed by Gomori's trichrome staining (Fig 4B). This was partially due to the increased thickness of the muscular wall, and it indicates areas of fibrotic replacement (Fig 4F).

### Significantly decreased density of intermuscular and intramuscular innervations accompanied by loss of neuronal cells in the infected group

**Fig 5:**
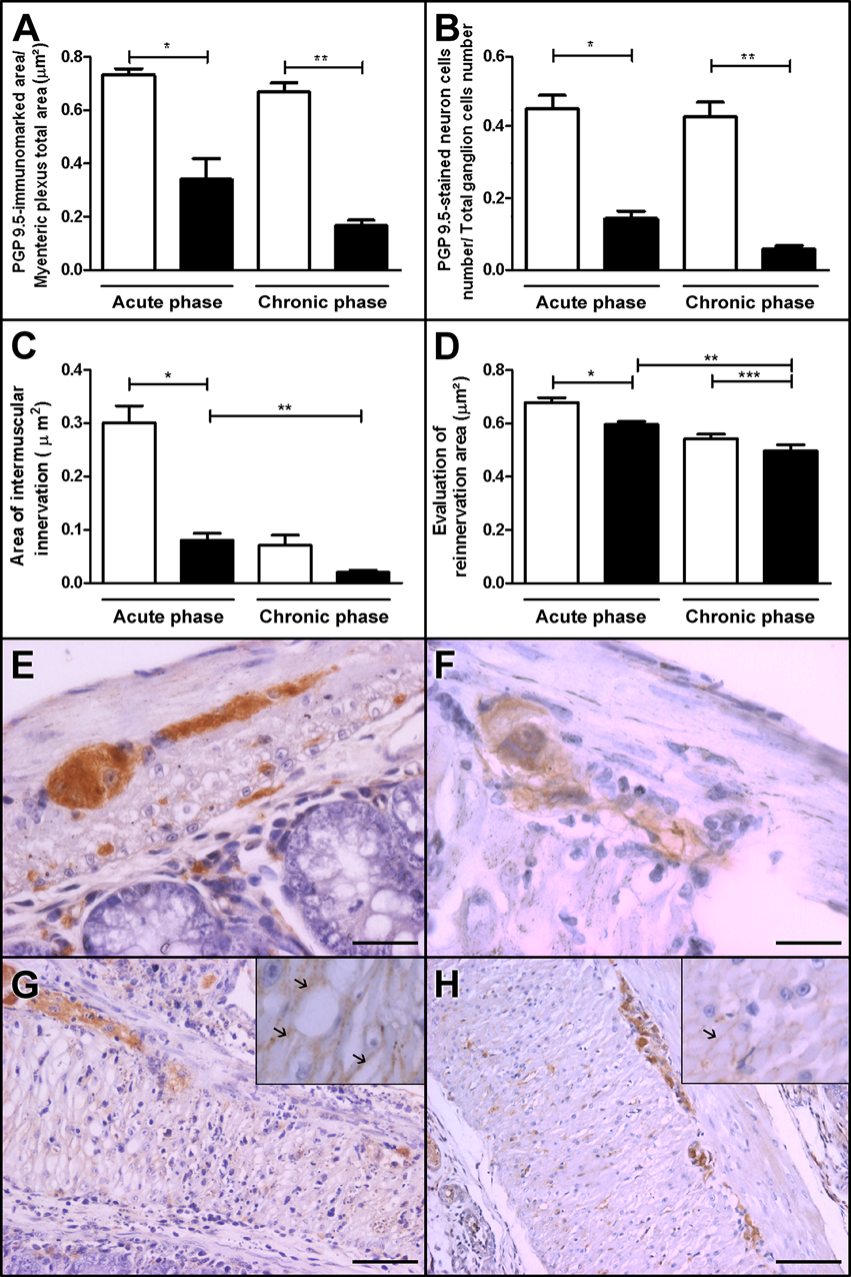
Intermuscular and intramuscular innervation of the colon and reinnervation phenomena in control and Swiss mice infected with 50,000 trypomastigotes of the Y strain of *T*. *cruzi* Y. (A, B, F and H) The myenteric plexus in the chronic-phase infected group showed decreased immunostaining for PGP 9.5. (A and F)—PGP 9.5-stained areas per myenteric plexus total area–, (B and F)—PGP 9.5-stained neuronal cells per total ganglion cell number compared with the (E control group. (C and H) n addition,in the chronic-phase infected group, intramuscular innervation decreased compared with (G) the acute-phase infected group (see arrows in insets) and (D) GAP 43 staining decreased compared with the acute-phase infected group. Values represent means ± SEM. *, **, ***, p<0.0001. N = 10 for each experimental and control group. The results represent two independent experiments. White: control group; black: infected group. E,F Magnification at 40× Scale bar represents 10 μm; G, H Magnification at 20x Scale bar represents 20 μm.

The acute-phase infected group presented a significant decrease in the density and number of neuronal cells per total ganglion cell number (density = 0.34 μm^2^; number of neuronal cells = 0.14) compared with the control group (density = 0.73 μm^2^; number of neuronal cells = 0.45; Fig 5A and 5B).

In the chronic phase, we observed a significant decrease in PGP 9.5-stained areas per myenteric plexus area in the infected group (0.17μm^2^; Fig 5A and 5F) compared with its control group (0.67 μm^2^; Fig 5E).

The number of PGP 9.5-stained neuronal cells per total ganglion cells decreased in the chronic-phase infected group (0.06 cells; Fig 5B and 5F) compared with its control group (0.43 cells; Fig 5E) as revealed by PGP 9.5-positive staining (loss of approximately 86% of the neuronal cells).

Importantly, there was a significant decrease in the area of intramuscular nerve fibers in the chronic-phase group (0.02 μm^2^; Fig 5C and 5H) compared with the acute-phase group (0.08 μm^2^; Fig 5G) and with the non-infected paired controls (0.07 μm^2^) (Fig 5C).

Our results indicate a discrete decrease in the GAP 43 expression in both acute and chronic phase; GAP 43 expression was investigated to evaluate a possible contribution of reinnervation on the density of intramuscular bundles (Fig 5D).

## Discussion

Our group proposes a novel model of long-term, chronic infection during CD by the treatment of animals in the acute phase (only after intense intestinal wall parasitism was detected). This treatment resulted in the absence of parasitemia and scarce parasitism detected by anti-*T*. *cruzi* immunohistochemistry in the chronic phase (15 m.a.i). The surviving animals were ELISA-positive for anti-*T*. *cruzi* IgG. They presented increased length and thickness of the colon wall (megadolicocolon) as well as muscular hypertrophy, fibrosis, loss of neurons in the myenteric plexus, and changes in intramuscular muscle innervation. These aspects mimic Chagasic megacolon in humans, and have been reproduced in an animal model for the first time in this study.

The Y strain used in our experiments is partially resistant to benznidazole [37]. It acts directly against circulating trypomastigotes and intracellular amastigotes, but its effectiveness depends on the length of treatment, dosing, and disease phase [38, 39, 40]. So, the animals that we treat with only a single dose of benznidazole at 500mg/kg of body weight improved their chances to survive but developed a chronic infection as we showed. The treatment with benznidazole allowed us to select the day 11 for euthanasia as the best period to detect parasites and their activity in the colon in the acute phase. This protocol ensured the control of parasitemia and the increase in survival, so that part of the animals that presented parasite-induced colonic wall inflammatory and degenerative changes reached the chronic phase.

Our results indicated that the parasites were detected in the colon a few days after the parasitemia peak. During this phase, the high mortality rate of infected animals compromised their survival and their subsequent progression to the chronic phase as already demonstrated [25]. In the present study, the peak of parasitemia occurred at day 8 in all infected animals, in accordance with the results of previous studies [21,22]. All untreated animals died between days 14 and 16.

In addition, in contrast to the results of other studies, our experimental design allowed us to assess the entire extension of the colon wall [34] of mice that were infected and euthanized at the 11^th^ d.a.i (acute phase) or treated with benznidazole and euthanized at the 15^th^ m.a.i (chronic phase). We used age-paired non-infected controls to interpret our results. The focal distribution of the lesion in the intestinal wall indicates biased sampling and measuring methods used by other authors [18,22].

*T*. *cruzi*-induced injury at the acute phase included parasitism, degeneration and necrosis of muscle fibers and resulted in intramuscular fibrosis and increased thickness of the colon wall which has also been described in patients with Chagasic megacolon, sometimes associated with mast cell function [41]. We did not notice significant differences in mast cell counts in the acute phase (control = 2.47 cells; infected = 1.73 cells; p = 0.67). However, there was a significant increase in the number of mast cells in the muscular layer in chronically infected mice compared with that in the controls (control = 0 cell; infected = 17 cells; p = 0.057).

The inflammatory foci had a transmural pattern (Fig 2C) but were intercalated with non-inflamed areas, a focal aspect also observed in the human disease. We did not notice parasitized neurons; however, there was visible damage to focal ganglion cells associated with intense inflammatory changes and parasitized glia (Fig 2E and inset) [30], which resulted in decreased morphometric parameters, as reported here. Previous studies with acute-phase rats (18 d.a.i.) also revealed a significant decrease in the number of neurons in the myenteric plexus, colon, and heart associated with intense inflammatory infiltration, focal leukocytes and lymphocytes in the muscular layer but without parasitized neurons [42].

On the basis of our extensive evaluation of the entire length of the colon, we noticed that the intense and focal destruction of myenteric ganglia in the acute phase was responsible for the decrease in the total myenteric area positive for PGP 9.5 (control = 0.67 μm^2^; infected = 0.17 μm^2^; Fig 5A, 5E and 5F).

The systematic observation of colon rolls obtained 15 m.a.i (chronic phase) evidenced only discrete parasitism in the submucosa and muscular layers in a few long-term infected animals. Most animals showed the presence of mononuclear cells in the muscular layer (Fig 2D) associated with perivascular infiltration, increased thickness of the vessel walls, and hypertrophy of the endothelium in the absence of patent parasitism. Similar findings have been described in Chagas’ disease and indicate vasculitis [43]. The mucous layer of 15-month-old control animals was slightly thinner than that of the controls euthanized at the 11^th^ d.a.i. This may be due to the ageing process or weight increase in the colon (Fig 1C). In non-inflamed areas, the neurons of myenteric and submucous ganglia appeared to be intact, despite the rare presence of inflammatory cells and/or amastigote nests in and around the myenteric plexus.

In this study, we used the anti-PGP 9.5 antibody to highlight the neural elements and performed a morphometric analysis of the intramuscular innervation, as shown in Fig 5A, 5B and 5C. The morphometric results indicated a significant decrease in the innervation density (expressed as PGP 9.5-stained area/total area of the myenteric plexus). We hypothesize that focal denervation may account for the progressive clinical course and explain not only the presence of asymptomatic patients but also the different levels of neuronal lesion [44] described in the human chronic phase of Chagas’ disease.

According to Koberle (1968) [45], dilatation and hypertrophy of the colon is initiated when the decrease in the number of ganglion cells exceeds the critical threshold of 55%. Our model also presents an opportunity to prove Koberle’s thesis of the acute-phase neuronal damage responsible for the neuronal loss (approximately 86%) and for the impairment of myenteric nerve function as one of the most likely mechanisms for development of Chagasic megacolon. In our opinion, in addition to the decreased number of neuronal cells in the myenteric plexus, one of our most original results is the significant decrease in the density of the intramuscular innervation (axonal density within the smooth muscle) in the chronic-phase group compared with its control. The activity of the smooth muscle of the GI tract is converted into coordinated peristalsis by the ganglion cells of the Aüerbach's plexus. Disturbance in the peristalsis leading to the lack of coordination of the wave-like forward motion causes stagnation, retention, and dilatation of hollow viscera. Because the distension of the muscle fibers is the primary cause of hypertrophy, continuous dilatation and hypertrophy lead to the well-known mega formations based on the disturbance of the intrinsic innervation of the organ [12,46]. The association between this pathogenetic mechanism and the inflammatory-induced intrinsic denervation can explain the natural history of mega formations in CD.

Our results indicate that the acute- and chronic-phase animals had a significant reduction in the density of the intermuscular innervation (loss of neurons of the myenteric plexus), particularly at the sites of intense inflammatory infiltrate (Fig 5A, 5B and 5C). In addition, the intramuscular network of nerve fibers decreased in the acute-phase infected group. In fact, our quantitative data suggest that the denervation rate was higher in the chronic phase (37.3%) compared with the acute phase (27.9%), indicating a possible progression over time and the persistence of continued damage to PGP 9.5-positive fibers even when inflammation was less intense (Fig 5C). One can speculate that the damaging mechanisms are continuously operating or even that differential mechanisms can be operating in each phase.

We also observed muscular layer hypertrophy in the chronic-phase infected animals (Fig 4A, 4C and 4D). Hypertrophy was observed in individual muscle cells of the circular layer, and interstitial fibrosis was also noted (Fig 4B, 4E and 4F). The increased area of Gomori’s trichrome staining in the chronic phase is also consistent with the results of previous studies showing moderate fibrosis [41] and decreased immunostaining of actin and myosin in muscle fibers [41]. These findings were also associated with the decreased density of the nerve fibers in the idiopathic megarectum [47] and with the decreased number of neuronal bodies observed in human mega formations [12,41,44] and in experimental acute Chagas’ disease [30].

Alterations to the intrinsic ENS were also observed in inflammatory bowel disease (IBD). Intestinal inflammation causes extensive growth of the smooth muscle cells in rat models of jejunitis and colitis [48], a process that contributes to the increased thickness of the intestinal wall observed in IBD and that can ultimately lead to intestinal stricturing in Crohn’s disease. The experimental model described here is a potential tool to study the mechanisms involved in the reduction of innervation and neuromuscular plasticity phenomena associated with inflammatory processes [49].

To compare our results with those of other authors who studied innervation and reinnervation in the ENS, we investigated the protein GAP 43, which is expressed in axonal growth cones. Despite its controversial role, GAP 43 has been shown to be the best marker for human intestinal nerve fibers compared with PGP and NSE [46]; in addition, it is a marker of reinnervation in the gut [50–52] and other organs [53]. The expression of GAP 43 in the myenteric plexus did not indicate significant reinnervation at the 15^th^ m.a.i. In fact, an average decrease in the GAP 43 expression was observed in the infected groups, although this expression was very irregular along the colonic segments (Fig 5D, 5F and 5H).

We reported previously that the denervation of the GI tract in the acute infection of C57BL/6 is dependent on NO production resulting from the activation of iNOS by IFN-γ in inflammatory foci [30]. Our results indicate that the anti-iNOS staining was diffuse and intense in the acute-phase infected animals compared with the chronic-phase animals (Fig 2I). It reinforces the role of NO in the battle against *T*. *cruzi* by murine macrophages in the acute phase of infection [54]. By contrast, NO can cause oxidative stress [55], which is harmful to the host and can cause neuronal damage to the myenteric plexus, and this mechanism offers an explanation for Koberle’s hypothesis [12].

Our long-term, chronic-phase histopathological results are unique and have not been revealed in other studies that evaluated the GI tract and the development of megacolon [21,22,25]. The characterization of this murine model of long-term, chronic infection leading to megacolon in CD may help elucidate the pathogenetic mechanisms of the chronic phase of this neglected disease, which causes high morbidity.
