# Correction: Enteric Neuronal Damage, Intramuscular Denervation and Smooth Muscle Phenotype Changes as Mechanisms of Chagasic Megacolon: Evidence from a Long-Term Murine Model of *Tripanosoma cruzi* Infection

**DOI:** 10.1371/journal.pone.0176224

**Published:** 2017-04-17

**Authors:** Camila França Campos, Silvia Dantas Cangussú, Ana Luiza Cassin Duz, Christiane Teixeira Cartelle, Maria de Lourdes Noviello, Vanja Maria Veloso, Maria Terezinha Bahia, Camila Megale Almeida-Leite, Rosa Maria Esteves Arantes

The genus name “*Trypanosoma*” is misspelled in the article title. The correct title is: Enteric Neuronal Damage, Intramuscular Denervation and Smooth Muscle Phenotype Changes as Mechanisms of Chagasic Megacolon: Evidence from a Long-Term Murine Model of *Trypanosoma cruzi* Infection. The correct citation is: Campos CF, Cangussú SD, Duz ALC, Cartelle CT, Noviello MdL, Veloso VM, et al. (2016) Enteric Neuronal Damage, Intramuscular Denervation and Smooth Muscle Phenotype Changes as Mechanisms of Chagasic Megacolon: Evidence from a Long-Term Murine Model of *Trypanosoma cruzi* Infection. PLoS ONE 11(4): e0153038. doi:10.1371/journal.pone.0153038
